# Abundant expression and functional participation of TRPV1 at Zusanli acupoint (ST36) in mice: mechanosensitive TRPV1 as an “acupuncture-responding channel”

**DOI:** 10.1186/1472-6882-14-96

**Published:** 2014-03-11

**Authors:** Shu-Yih Wu, Wei-Hsin Chen, Ching-Liang Hsieh, Yi-Wen Lin

**Affiliations:** 1 Graduate Institute of Acupuncture Science, College of Chinese Medicine, China Medical University, 91 Hsueh-Shih Road, Taichung 40402, Taiwan; 2 Graduate Institute of Biotechnology, College of Agriculture And Natural Resources, National Chung Hsing University, 250 Kuo Kuang Rd, Taichung 402, Taiwan; 3 Acupuncture Research Center, China Medical University, 91 Hsueh-Shih Road, Taichung 40402, Taiwan; 4 Graduate Institute of Integrated Medicine, College of Chinese Medicine, China Medical University, 91 Hsueh-Shih Road, Taichung 40402, Taiwan; 5 Department of Chinese Medicine, China Medical University Hospital, 2 Yuh Der Road, Taichung 40402, Taiwan

**Keywords:** Acupuncture, TRPV1, TRPV4, ASIC3, Mechanotransduction, Calcium wave propagation

## Abstract

**Background:**

Acupuncture is a therapy that involves applying mechanical stimulation to acupoints using needles. Although acupuncture is believed to trigger neural regulation by opioids or adenosine, still little is known about how physical stimulation is turned into neurological signaling. The transient receptor potential vanilloid receptors 1 and 4 (TRPV1 and TRPV4) and the acid-sensing ion channel 3 (ASIC3) are regarded as mechanosensitive channels. This study aimed to clarify their role at the Zusanli acupoint (ST36) and propose possible sensing pathways linking channel activation to neurological signaling.

**Methods:**

First, tissues from different anatomical layers of ST36 and the sham point were sampled, and channel expressions between the two points were compared using western blotting. Second, immunofluorescence was performed at ST36 to reveal distribution pattern of the channels. Third, agonist of the channels were injected into ST36 and tested in a mouse inflammatory pain model to seek if agonist injection could replicate acupuncture-like analgesic effect. Last, the components of proposed downstream sensing pathway were tested with western blotting to determine if they were expressed in tissues with positive mechanosensitive channel expression.

**Results:**

The results from western blotting demonstrated an abundance of TRPV1, TRPV4, and ASIC3 in anatomical layers of ST36. Furthermore, immunofluorescence showed these channels were expressed in both neural and non-neural cells at ST36. However, only capsaicin, a TRPV1 agonist, replicated the analgesic effect of acupuncture when injected into ST36. Components of calcium wave propagation (CWP, the proposed downstream sensing pathway) were also expressed in tissues with abundant TRPV1 expression, the muscle and epimysium layers.

**Conclusions:**

The results demonstrated mechanosensitive channel TRPV1 is highly expressed at ST36 and possibly participated in acupuncture related analgesia. Since CWP was reported by other to occur during acupuncture and its components were shown here to express in tissues with positive TRPV1 expression. These findings suggest TRPV1 might act as acupuncture-responding channel by sensing physical stimulation from acupuncture and conducting the signaling via CWP to nerve terminals. This study provided a better understanding between physical stimulation from acupuncture to neurological signaling.

## Background

Acupuncture is an ancient therapy that gained worldwide acknowledgment in recent decades [1]. It involves inserting needles into acupoints followed by manual manipulation (manual acupuncture, MA) or electrostimulation (electroacupuncture, EA) to induce its therapeutic effect in epilepsy, [2] stroke, [3] and pain treatment [4-6]. To confirm its efficacy, clinical studies have shown its benefits, particularly in pain management [7-13].

Although acupuncture efficacy is demonstrated in clinical trials, little is known about acupuncture mechanism. Many authors have reported activation of endogenous opioid peptide related antinocipeptive pathways during acupuncture, which involves the arcuate nucleus, the periaqueductal gray, the nucleus raphe magnus, and the descending inhibitory pathways [14-17]. Recently, Goldman *et al.* demonstrated localized ATP release at acupoints after MA [18,19]. ATP is then metabolized to antinociceptive adenosine by prostatic acid phosphatase in muscles, resulting in analgesia. This finding was further augmented by reduced acupuncture analgesia in adenosine A1 receptor knockout mice. These results demonstrated that acupuncture is related to local ATP release and subsequent neural regulation. However, it remains unknown how mechanostimulation from acupuncture induces ATP release and neural stimulation.

Considering acupuncture involves applying mechanostimulation to acupoints by needles, we suggest that mechanosensitive channels are involved in the reception of mechanostimulation. The transient receptor potential vanilloid receptors 1 and 4 (TRPV1 and TRPV4) and the acid-sensing ion channel 3 (ASIC3) are mechanosensitive channels related to local ATP release in various tissues [20-25]. They are structurally membrane channel proteins permeable to cations, as sodium and calcium, after stimulated mechanically. After stimulation, the opened mechanosensitive channels would lead to influx of cation and increase membrane potential. When this happens at cell membrane of a neuron, action potential occurs. Gadolinium is a nonselective mechanosensitive channel blocker that also blocks TRPV1, TRPV4, and ASIC3. It’s known that the appliance of gadolinium greatly reduce mechanically-activated current of neuron in vitro. Interestingly, MA effects are blocked by gadolinium when it was applied systemically to rats before MA [26-28]. Taking concern their mechanosensitivity and the role played in stimulation-induced ATP release, it is highly possible that these channels participate in acupuncture sensing.

It is noteworthy that local ATP released is related to the intercellular purinergic signaling called calcium wave propagation (CWP). Once activated by extracellular ATP via purinergic P2Y receptors, the stimulated cells are then processed through intracellular calcium signaling, resulting in ATP release by hemichannels (e.g., pannexin 1 or connexin 43). ATP released from cells then stimulates purinergic receptors in nearby cells in a paracrine manner and causes both intracellular calcium signaling and ATP release. This chain-like process can continue for a certain distance. The phenomenon is universal and is reported among glia, [29] salivary glands, [30] nephrons, [31] fibroblasts, [32] keratinocytes, [33] etc. In subepithelial fibroblasts of villi [32] and keratinocytes [33], it has been proposed that non-neural cells respond to mechanostimulation by ATP release and send signals to nerve terminals via CWP. The occurrence of CWP during acupuncture in non-neural cells was recently reported [34]. As mechanosensitive channels are related to ATP release, it is rational to believe signaling is conducted via CWP to nerve terminals after stimulation of non-neural cell by acupuncture.

In this study, we hypothesized that during manual acupuncture, mechanosensitive channels participate in sensing physical stimulation from acupuncture and conducting the signaling via CWP to nerve terminals. This was first demonstrated by the abundant mechanosensitive channels expression at neural and non-neural tissues of Zusanli acupoint (ST36) followed by the replication of the acupuncture analgesic effect after injecting agonist of the channels into ST36. Finally, this study demonstrated abundant expression of CWP components (pannexin 1, connexin 43, P2Y1, and P2Y2) in tissues with positive mechanosensitive channel expression at ST36, which implies the occurrence of CWP after channel stimulation. The results of this study provide a better picture of the interface between physical stimulation by acupuncture and biological signaling to the nervous system.

## Methods

### Animal

Experiments were carried out on ICR mice (aged 8 to 12 weeks) purchased from BioLASCO Co., Ltd, Taipei, Taiwan. After arrival, 12 hr light–dark cycle with sufficient water and food were given. All procedures were approved by the Institute of Animal Care and Use Committee of China Medical University (permit No. 101-116-N) and were in accordance with *Guide for the use of Laboratory Animals* by National Research Council [35] and with the ethical guideline of the International Association for the study of pain [36]. The number of animal used and their suffering were minimized.

### Inflammatory pain model and behavioral tests

To generate an inflammatory pain model, mice were anesthetized with 2% isoflurane, and 20 μL CFA (1:1 mixture of saline with complete Freund’s adjuvant; Sigma-Aldrich, St. Louis, MO, USA) was subcutaneously injected into the right hind paw [37]. MA and agonist injections were given once on day 3 (D3).

A thermal hyperalgesia test was performed using Hargraves’ test IITC analgesiometer (IITC Life Sciences, Woodland Hills, CA, USA). The test was performed on day 0 before CFA injection (D0), on day 3 before intervention (MA or drug injection) (D3 pre), on day 3, approximately 60 min after intervention (D3 post), and on D4, which was 24 h after intervention. To perform the test, a radiant heat source was focused on the right hind paw, and withdrawal latency was determined as the time taken for paw removal. During each tested time point, five repeated tests were conducted, and the average was calculated. To avoid damage to tissues, a resting interval of at least 4–5 min was set between tests, and the maximum time of heat focus was 20 s. To minimize the effect of isoflurane, 2% isoflurane was given 60 min before each test, for approximately 30 min with or without intervention. We divided the averaged withdrawal latency of every time point by the averaged latency recorded on D0, as the final withdrawal latency ratio, to minimize individual variance among mice. For comparisons between groups, the change of ratio was calculated by simply subtracting the ratio recorded on D3 before intervention from the ratio of selected time points.

### Manual acupuncture

After anesthesia with 2% isoflurane, MA was performed by inserting a stainless steel acupuncture needle (diameter, 0.16 mm; length, 7.5 mm; Shinlin CO., Ltd, Tianjin, China) into ipsilateral Zusanli acupoint (ST36) of the inflamed limb. The location of ST36 is approximately 4 mm below and 1–2 mm lateral to the midpoint of the knee in mice. An ipsilateral nonacupoint located around the midpoint of the superior edge of the gluteus maximus muscle was selected as the sham. ST36 was selected because of its well-recognized analgesia effect in mouse pain models, and the sham point was used because of the relative scarce acupoints located in the region [19,38,39]. This sham point was also suitable because it is located between two meridians in the region, the urinary bladder and gallbladder meridians, and is a distant from the frequently used acupoint GB40 [39]. To ensure an insertion depth of 3 mm, a piece of tape was stuck to the needle, leaving only space for manipulation and a needle tip of 3 mm. During acupuncture, the tape was used as a guide and the needle was twisted 360° anticlockwise, then back for one twist at a speed of approximately 1 turn/s. A protocol of two twists every 5 min for duration of 30 min was followed as described by Goldman *et al*. [19]. Tests on the needle group were performed by only inserting a needle at ST36, without any twisting. Tests on the sham group were performed by manipulation as MA at the sham point. The first behavioral test after MA was performed 50–70 min after acupuncture, which represented an average of 60 min after MA.

### Drugs and injection method

The TRPV1 agonist capsaicin (0.5%; Sigma-Aldrich, St. Louis, MO, USA) was dissolved in 5% ethanol, 5% Tween-20, and 90% saline. The concentration of capsaicin was selected based on the report by Gear *et al.*: a concentration of 0.5% yielded the maximal noxious stimulus-induced analgesia [40]. The TRPV4 agonist GSK1016790A (Sigma-Aldrich, St. Louis, MO, USA) was given at concentrations of 0.02% (almost saturated in the vehicle used), 0.01%, and 0.001%, respectively, in 5% DMSO, 5% Tween-20, and 90% saline. These concentrations were chosen after calculating the ratio of the concentration used and the half-maximal effective concentration (EC50) provided by the drug company to achieve a ratio of GSK1016790A similar to the ratio used for capsaicin injections, because capsaicin was demonstrated to replicate analgesia according to the results of this study. Acidified saline solutions (pH 5, 4, and 3) were used as agonists of ASIC3. They were prepared using 0.01 M 2-[*N*-morpholino] ethanesulfonic acid (MES) dissolved in saline and pH-adjusted with 0.1 M HCl or NaOH. These pH values were selected because Jerzy Karczewski *et al*. [41]. reported that APETx2, an ASIC3-specific antagonist, reduced mechanical hypersensitivity in a rodent acid-induced muscle pain model created by repeated injection of pH 4 saline . They concluded that ASIC3 is the major sensing component after injection of pH 4 saline into muscle. A pH 7.4 vehicle control was prepared as an injection fluid without drugs.

After anesthesia with 2% isoflurane, 10 μL of the drug or vehicle were injected 3 mm deep at ST36 or the sham acupoint (located as described in the MA method). GSK1016790A and acidified saline were injected into ST36 only. The first behavioral test after the injection was performed 50–70 min later, representing an average of approximately 60 min. All animals were grossly normal during behavioral tests.

### Tissue sampling and western blot analysis

**Figure 1:**
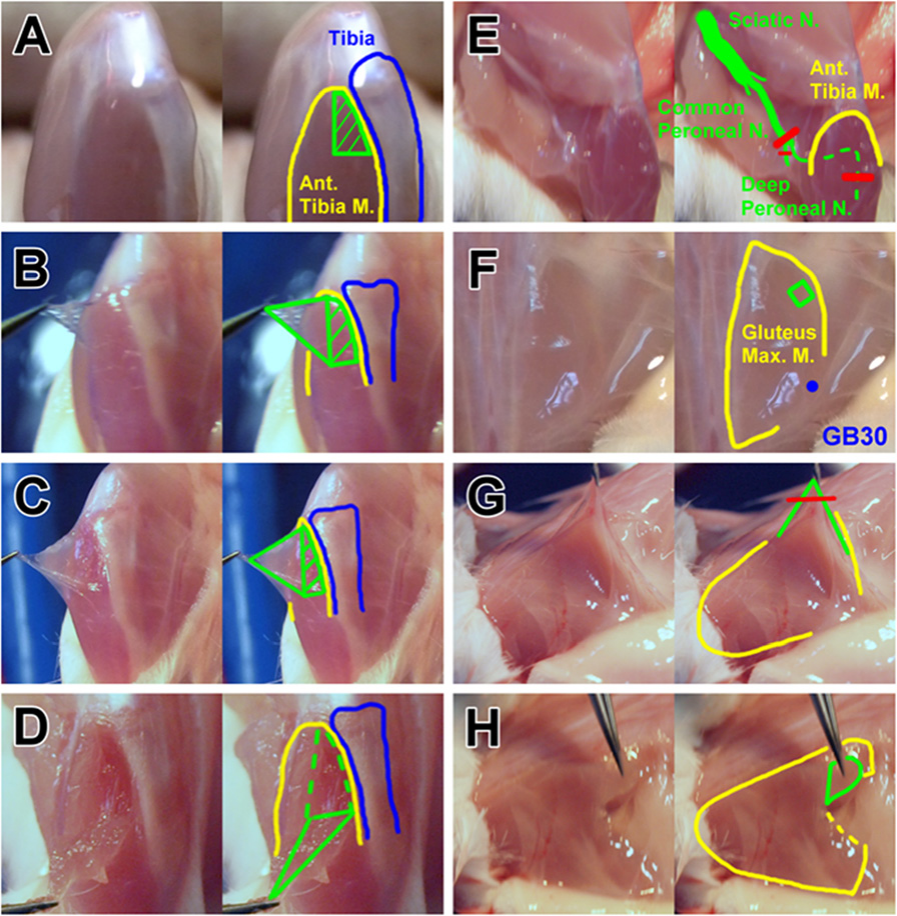
**Tissue sampling from ST36 and the sham point. (A)** ST36 was located 4 mm below and 1–2 mm lateral to the midpoint of the knee, and **(F)** the sham point was defined at the midpoint of the superior edge of the gluteus maximus muscle. At ST36, **(B)** subcutaneous loose connective tissue (ScLCT), **(C)** epimysium, **(D)** muscle, and **(E)** nerve were obtained (green areas). At the sham point, only **(G)** ScLCT and **(H)** muscle were obtained because of technical issues. Margin of the muscle (yellow), bone (blue), and a frequently used acupoint near the region (GB30).

Mice were initially anesthetized with an overdose of choral hydrate and intracardially perfused with saline. Samples of subcutaneous loose connective tissue (ScLCT), epimysium, muscle tissue, and the deep peroneal nerve were collected at ST36. After skin dissection, the ScLCT (with the appearance of a ground-glass-like sheet) overlying ST36 was pulled up lightly using microforceps and retrieved with a microscissor (Figure 1B). Subsequently, microforceps were used to gently and bluntly dissect the cut edge of the ScLCT to clear the remaining ScLCT. The epimysium, a whitish membrane overlying the anterior tibia muscle, was identified. Because ST36 is located at the medial side of the anterior tibia muscle, a vertical incision on the midline of the muscle belly was made and another along the lateral border of the tibia. Subsequently, the upper portion of the epimysium was taken (approximately 20% of the tibia length) (Figure 1C). Any muscle tissue remaining on the epimysium was tried to be separated. Muscle tissue located directly under the sampling field of the epimysium was gathered (Figure 1D). To dissect the deep peroneal nerve, first the sciatic nerve was identified at the mid-thigh level and then dissection was made along the track of the nerve to identify the common and deep peroneal nerves. The upper quarter of the deep peroneal nerve and part of the common peroneal nerve, near the fibula, was cut as a nerve sample (Figure 1E). A similar sampling method was applied at the sham point (Figure 1G and H); however, the epimysium and nerve tissue were not retrieved because of technical difficulties.

Sampled proteins were prepared by adding lysis buffer containing 50 mM Tris–HCl pH 7.4, 250 mM NaCl, 1% NP-40, 5 mM EDTA, 50 mM NaF, 1 mM Na_3_VO_4_, 0.02% NaN_3_, and 1× protease inhibitor cocktail (AMRESCO, Solon, OH, USA) to samples. They were then homogenized using a Bullet Blender homogenizer (Next Advance, NY, USA). The extracted proteins (30 μg/sample, as assessed using the BCA protein assay) were subjected to 8% SDS-Tris glycine gel electrophoresis and transferred to a PVDF membrane. The membrane was blocked with 5% nonfat milk in TBS-T buffer (10 mM Tris pH 7.5, 100 mM NaCl, and 0.1% Tween-20) and incubated with the appropriate antibody overnight at 4°C in TBS-T with 1% bovine serum albumin (BSA). The primary antibodies used were anti-TRPV1 (1:1000), anti-TRPV4 (1:1000), anti-ASIC3 (1:500), and anti-P2Y1 (1:500) from Alomone, Jerusalem, Israel; anti-pannexin 1 (1:125) and anti-connexin 43 (1:500) from Invitrogen, New York, USA; anti-PGP9.5 (1:250) and anti-P2Y2 (1:500) from Abcam, Cambridge, MA, USA; and anti-α-tubulin (1:1000) from Santa Cruz, Dallas, Texas, USA. A peroxidase-conjugated anti-rabbit or anti-mouse (1:10,000) antibody (Jackson ImmunoResearch Laboratories, West Grove, PA, USA) was used as a secondary antibody. The bands were enhanced using a chemiluminescence kit (T-Pro Biotechnology, New Taipei, Taiwan) and visualized with LAS-3000 Fujifilm (Fuji Photo Film Co. Ltd, Tokyo, Japan) or Fusion-SL (Vilber Lourmat, France). The intensities of specific bands were quantified with the NIH ImageJ software (Bethesda, MD, USA). The ratios of proteins were obtained by dividing the intensities of target proteins by the intensity of α-tubulin from the same sample. The calculated ratios were then adjusted by dividing the ratios from the same comparison group to those of the control (muscle or ScLCT from the sham point). Note that the epimysium is a histologically dense connective tissue but shares similar cell types with ScLCT (mostly fibroblasts). The most important difference between the two is cell density; thus, they were placed in the same comparison group after normalization.

### Immunofluorescence

Animals were anesthetized with an overdose of choral hydrate and intracardially perfused with saline. Two cutting sections, located 5 mm above and below ST36, were made vertical to the tibia bone. The samples collected were decalcified in 13% EDTA (pH 7.3) for 3 days and then placed in 30% sucrose overnight and embedded in OCT at −20°C the following day. Frozen sections were cut (30 μm) and placed on glass microslides coated with APS. Subsequently, the sections were postfixed in 4% paraformaldehyde for 3 min and incubated in blocking solution containing 3% BSA, 0.1% Triton X-100, and 0.02% NaN_3_ in PBS for 2 h at room temperature. After blocking, sections were incubated with the appropriate primary antibodies in blocking solution at 4°C overnight. Note that sections were incubated in blocking solution without a primary antibody for the negative control. The primary antibodies used were: anti-TRPV1 (1:500), anti-TRPV4 (1:500), and anti-ASIC3 (1:400) from Alomone; and anti-PGP9.5 (1:200) from Abcam. The secondary antibody was a goat anti-rabbit (1:500) antibody (Molecular Probes, Carlsbad, CA, USA). Slides were mounted with cover slips and visualized using a fluorescence microscope (CKX41 with an Olympus U-RFLT50 Power Supply Unit; Olympus, Tokyo, Japan). During microscopic observation, ST36 was defined as described below. First, an imaginary line connecting the tibia and the fibula was set. The point located at the medial one third of the line was identified and ST36 was defined as the projection from that point to the dermomuscular junction of the anterior tibia muscle.

### Statistical analysis

All statistic data are presented as the mean ± standard error of the mean. Statistical significance was tested using Mann–Whitney Rank Sum Test (*P* < 0.05 was considered statistically significant) with SigmaStat for windows version 3.5 (Systat Software Inc, Chicago, USA).

## Results

### Manual acupuncture had an analgesic effect at ST36 but not at the sham point

**Figure 2:**
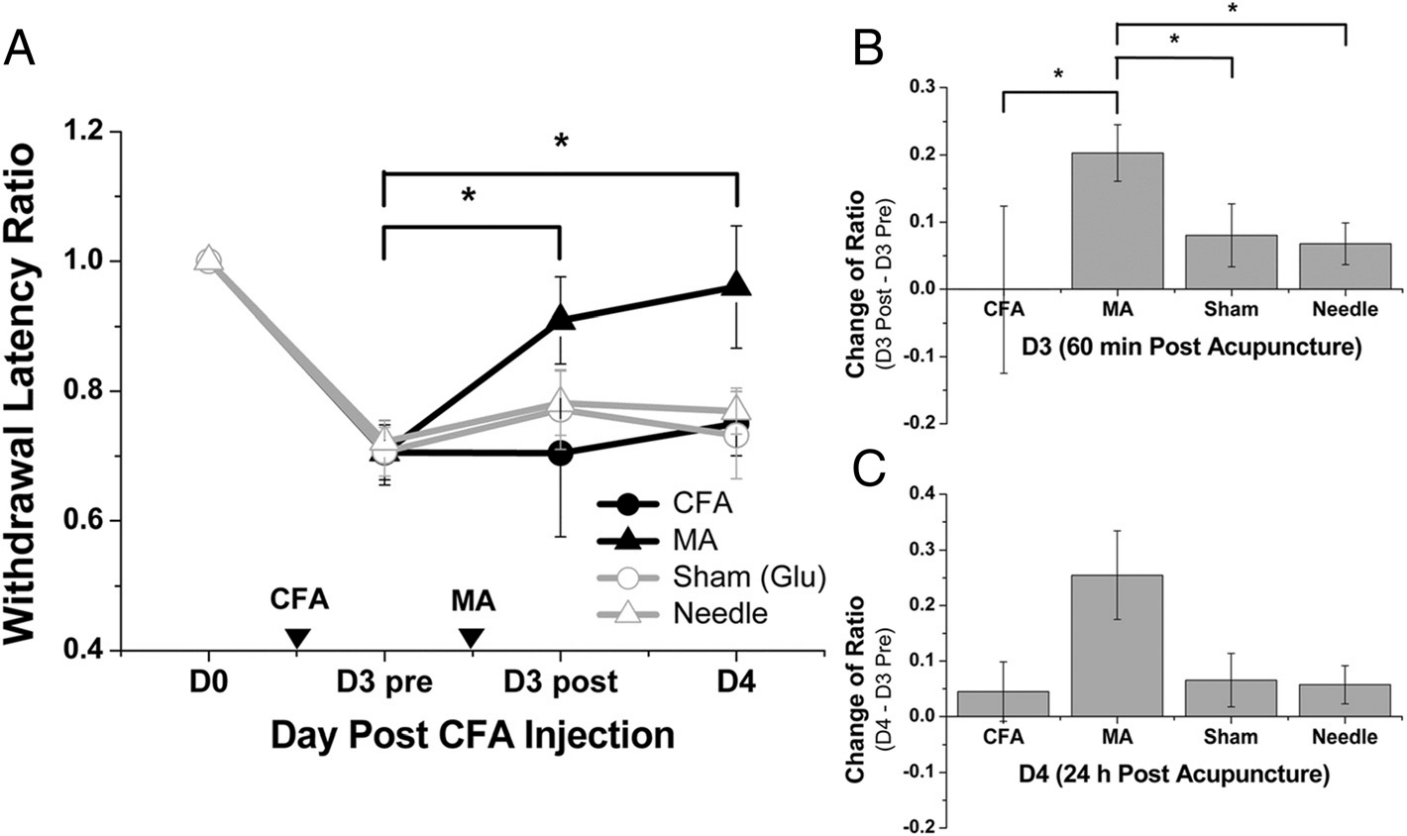
**Manual acupuncture (MA) at ST36 yielded an analgesic effect in mouse CFA inflammatory pain model.** On day 3 (D3) after CFA injection, MA, sham treatment (Sham), or needling without twisting (Needle) were administered at ST36 or sham point. Hargraves’ thermal test was performed before CFA injection (D0), on D3 preintervention (D3 pre), on D3 postintervention (D3 post, 60 min after intervention), and on D4 (24 h after intervention). **(A)** MA at ST36 yielded an analgesic effect in within-group tests on D3 post (*P* < 0.05) and on D4 (*P* < 0.05). **(B)** Between-group tests showed that MA yielded significant differences compared with CFA (*P* < 0.05), Sham (*P* < 0.05), and Needle (*P* < 0.05) groups on D3 post. **(C)** No significant differences were observed on D4 in between-group tests (*P < 0.05, Mann–Whitney rank sum test, *n* = 9–14). Data are means ± S.E.M.

Before demonstrating the existence of mechanosensitive channels at ST36, it is important to make sure that ST36 and the sham point defined were truly a functional acupoint and a nonfunctional sham point, respectively. First, MA at the defined ST36, but not at the sham point, effectively relieved thermal hyperalgesia in Hargraves’ thermal test was demonstrated in a mouse CFA inflammatory pain model (Figure 2A-C).

The results showed that 60 min after MA at ST36 on D3, thermal hyperalgesia was significantly reduced compared with that observed before MA because the withdrawal latency ratio increased from 0.71 ± 0.04 to 0.91 ± 0.07 (*P* < 0.05; Mann–Whitney rank sum test) (Figure 2A). On D4, 24 h after MA, the withdrawal latency ratio in the MA group was 0.96 ± 0.09; this remained significant compared with the ratio observed before MA on D3 (*P* < 0.05). The change in latency ratios on D3 were calculated among the tested groups on D3 and showed as follow: the MA group showed a change of 0.20 ± 0.04, the CFA group (without intervention) exhibited a change of 0.00 ± 0.12, the sham group (acupuncture manipulation at the sham point) showed a change of 0.08 ± 0.05, and the needle group (insertion at ST36 without manipulation) showed a change of 0.07 ± 0.03 (Figure 2B). Significant differences regarding change of ratio were observed only between the MA group and the remaining three groups (*P* < 0.05; Mann–Whitney rank sum test). However, there was no significant difference between the change of ratio among any of the groups on D4 (Figure 2C). The difference in the results of within-group comparisons and between-group comparisons on D4 was because of a relatively larger variation on D4. This may reflect a variation in the MA therapeutic duration between individual mice and agitation of some tested mice after three successive behavior tests. The observation that the analgesic effect was only observed in the MA group implies that acupuncture analgesia can only be induced on acupoints and that manipulation is vital for the effect. Moreover, the locations of the functional acupoint ST36 and the nonfunctional sham point were as defined.

### Mechanosensitive channels were abundantly expressed at ST36

After assuring that the locations of the functional ST36 and the nonfunctional sham point were as defined, samples from the two points was gathered to determine if there were differences in the expression of the mechanosensitive channels TRPV1, TRPV4, and ASIC3 using western blotting. Neural tissue (Ner, deep peroneal nerve), subcutaneous loose connective tissue (ScLCT), epimysium (Epi), and muscle (Mus) were obtained from ST36. Because of technical difficulties, only subcutaneous loose connective tissue and muscle were obtained from the gluteus sham point. Notably, although epimysium is a dense connective tissue, the tissue share similar cellular components with the ScLCT (mostly fibroblasts), albeit with different cell densities. Therefore, after normalization, they were still placed in the same comparison group.

**Figure 3:**
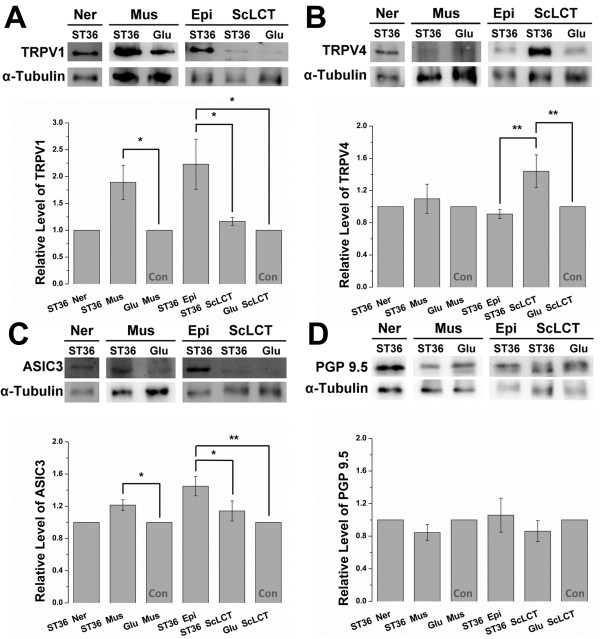
**Western blotting showed TRPV1, TRPV4, and ASIC3 were abundantly expressed at ST36.** Tissues were sampled from nerve (Ner), muscle (Mus), epimysium (Epi), and subcutaneous loose connective tissue (ScLCT) of ST36. Only muscle and ScLCT were obtained from the sham point because of technical issues and were both used as control (Con) in separate comparison groups. **(A, B, C, and D)** TRPV1, TRPV4, ASIC3, and PGP 9.5 (a pan-neural marker) were expressed in nerve, muscle, and connective tissue. **(A)** TRPV1 was abundantly expressed at ST36 muscle (*P* < 0.05) compared with sham, and ST36 epimysium exhibited higher expression compared with ST36 ScLCT (*P* < 0.05) and sham ScLCT (*P* < 0.05). **(B)** TRPV4 was abundantly expressed at ST36 ScLCT compared with ST36 epimysium (*P* < 0.01) and sham ScLCT (*P* < 0.01). **(C)** ASIC 3 exhibited higher expression at ST36 muscle compared with the sham (*P* < 0.05), and at ST36 epimysium compared with ST36 ScLCT (*P* < 0.05) and sham ScLCT (*P* < 0.01). **(D)** PGP 9.5 was not significantly expressed at ST36 or the sham point, which indicated an absence of differences in nerve distribution in these two points. The relative level of target proteins was calculated by dividing the ratio of targeted proteins (targeted protein/α-tubulin) by the ratio of control in the comparison group. ST36 epimysium, ST36 ScLCT, and sham ScLCT were placed in the same comparison group because their cellular components were similar (mostly fibroblasts), even though their cell densities were different (*P < 0.05, **P < 0.01; Mann–Whitney rank sum test; TRPV1, *n* = 6; TRPV4, *n* = 7; ASIC3, *n* = 7–10; PGP 9.5, *n* = 9). Data are means ± S.E.M.

The results of this experiment demonstrated that all three channels were positively expressed in neural tissue. TRPV1 was abundantly expressed at ST36 muscle (1.89 ± 0.32-fold over the sham, *P* < 0.05; Mann–Whitney rank sum test) compared with the sham. TRPV1 was abundantly expressed at ST36 epimysium compared with sham ScLCT (2.23 ± 0.47-fold, *P* < 0.05) and ST36 ScLCT (2.23 ± 0.47 vs. 1.17 ± 0.07, *P* < 0.05) (Figure 3A). TRPV4 was expressed in muscle, but no difference was found between ST36 muscle and the sham (1.10 ± 0.18-fold vs. the sham). A significant difference was observed between ST36 ScLCT and ST36 Epi (1.44 ± 0.20 vs. 0.91 ± 0.06, *P* < 0.01) and between ST36 ScLCT and sham ScLCT (1.44 ± 0.20-fold, *P* < 0.01) (Figure 3B). ASIC3 was more abundant in ST36 muscle than in the sham (1.21 ± 0.07-fold, *P* < 0.05) and was predominantly expressed in ST36 epimysium compared with ST36 ScLCT (1.45 ± 0.12 vs. 1.14 ± 0.12, *P* < 0.05) and sham ScLCT (1.45 ± 0.12-fold, *P* < 0.01) (Figure 3C).

To verify if the expression patterns observed could be attributed to differences in neural distribution or differences in the expressed levels of channels, the expression pattern of the pan-neuronal maker PGP 9.5 was tested. PGP 9.5 was expressed in neural, muscle, epimysium, and ScLCT tissues. This demonstrated neural innervation in the anatomical layers. However, there was no difference in expression in muscle or connective tissue comparing ST36 with the sham point (Figure 3D). This indicated that there was no difference in neural distribution between ST36 and the sham point. The abundance in TRPV1, TRPV4, and ASIC3 shown at ST36 was the result of an increased number of channels expressed in the anatomical layers.

### Mechanosensitive channels were expressed in neural and non-neural cells

**Figure 4:**
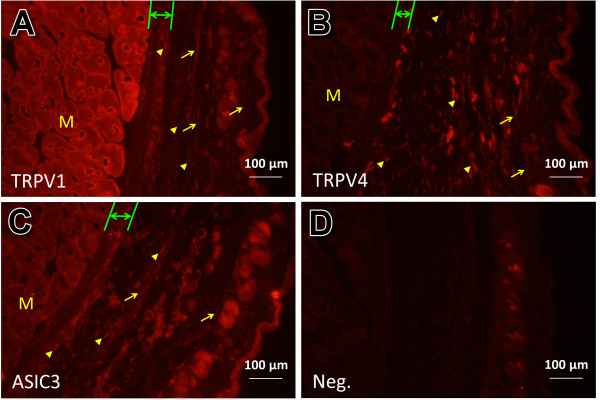
**Immunofluorescence of TRPV1, TRPV4, and ASIC3 at ST36.** TRPV1 **(A)**, TRPV4 **(B)**, and ASIC3 **(C)** were primarily expressed in muscle fibers (M), nerve (arrow), and subcutaneous cells (arrowhead) (as assessed using immunofluorescence). Immunofluorescence results correlated with the patterns observed in western blots. TRPV1 and ASIC3 showed evident expression in muscle and epimysium (between green lines), and TRPV4 was evident in subcutaneous cells. Note that stronger fluorescence was observed at the cell margin in muscles, which is in accordance with their role as membrane channels. **(A)** In particular, TRPV1 was expressed more strongly at a very thin layer located beneath the epimysium. Scale bars are as shown. **(D)**, negative controls (no primary antibody).

The experiments described above showed that TRPV1, TRPV4, and ASIC3 were abundantly expressed in the anatomical layers of ST36. Nonetheless, the results of western blotting did not reveal the histological expression of channels. Therefore, immunofluorescence at ST36 was performed (Figure 4A–D). First, ST36 location was microscopically defined. The microscopic section showed that all three channels were expressed in subcutaneous nerve fibers (arrow) (Figure 4A, B, and C). They were also expressed in muscle, particularly in the cell membrane (higher expression at the margin of muscle fibers). This correlates with their role as membrane channels. Higher fluorescence was observed in muscle fibers labeled for TRPV1. In contrast, TRPV4 showed a relatively lower expression. This difference in expression is consistent with the findings of western blotting because TRPV1 exhibited the highest relative level of expression. Interestingly, only for TRPV1, a very thin layer at the margin of the muscle beneath the epimysium which exhibited even higher expression was discovered (Figure 4A). This is rather interesting because acupuncture sensation (de-qi) is stronger just after the needle tip enters the perimuscular fascia (epimysium) [42].

All three channels showed positive expression in the cells of the epimysium (between green lines) (Figure 4A, B, and C) and subcutis (arrowhead). In accordance with the results of western blotting, expression in subcutaneous cells seemed relatively evident for TRPV4. The positive cells observed in the epimysium and subcutis were considered mainly as fibroblasts, taking into account that fibroblasts are the principal cell in connective tissue [43] and that TRPV1 [44], TRPV4, [45,46] and ASIC3 [47,48] are reported to express in fibroblast. However, these positive cells in the epimysium and subcutis could be cells other than fibroblasts, such as mast cells. The immunofluorescence experiments showed that TRPV1, TRPV4, and ASIC3 were expressed in neural and non-neural cells (muscle cells and maybe fibroblasts).

### Injection of the TRPV1 agonist capsaicin into ST36 replicated the acupuncture-like analgesic effect

Next, we aimed to determine whether the activation of these channels would produce an acupuncture-like analgesic effect. This was achieved by injecting 10 μL of agonist into ST36 and testing in the mouse CFA inflammatory pain model.

**Figure 5:**
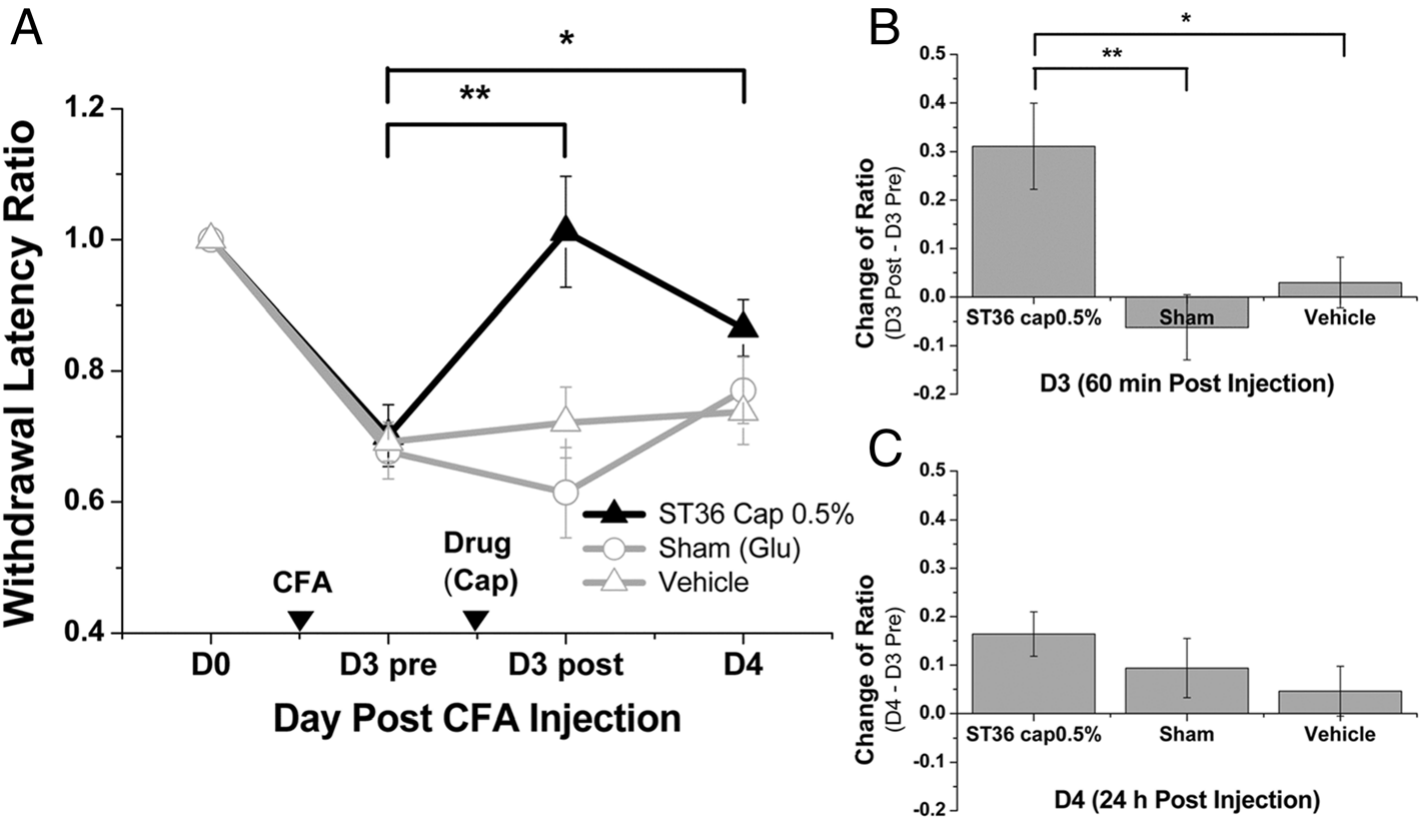
**TRPV1 agonist (capsaicin) injection at ST36 replicated the analgesic effect of acupuncture.** The behavioral test design was similar to that of MA, with the exception that capsaicin was injected at a concentration of 0.5% and a volume of 10 μL into ST36 or sham point. **(A)** In within-group tests, capsaicin injections into ST36 showed significant effect on D3 post (*P* < 0.01) and on D4 (*P* < 0.05). **(B)** In between-group tests, capsaicin injections at ST36 yielded significant differences compared with injections at the sham point (*P* < 0.01) and vehicle injections at ST36 (*P* < 0.05) on D3 post. **(C)** There were no significant differences in between-group tests on D4. Within-group and between-group tests were designed similar to the MA behavioral test (*P < 0.05, **P < 0.01; Mann–Whitney rank sum test, *n* = 11–13). Data are means ± S.E.M.

In the case of TRPV1, 0.5% capsaicin was injected that resulted in an analgesic effect similar to that of MA (Figure 5A–C). On D3, the withdrawal latency ratio increased from 0.70 ± 0.05 before injection to 1.01 ± 0.08 after injection (*P* < 0.01; Mann–Whitney rank sum test) (Figure 5A). The antinociceptive effect of capsaicin injection persisted on D4, with a ratio of 0.87 ± 0.04 (*P* < 0.05 compared with the ratio recorded on D3 pre). The comparison between groups on D3 revealed that change of ratio was significantly different between the capsaicin and sham groups (injection of 10 μL 0.5% capsaicin into the sham point) (0.31 ± 0.09 vs. −0.06 ± 0.07, *P* < 0.01) (Figure 5B). Similarly, the comparison of the capsaicin and vehicle groups showed significant differences (0.31 ± 0.09 vs. 0.03 ± 0.05, *P* < 0.05). However, no significant difference in change of ratio was found between groups on D4 (Figure 5C).

**Figure 6:**
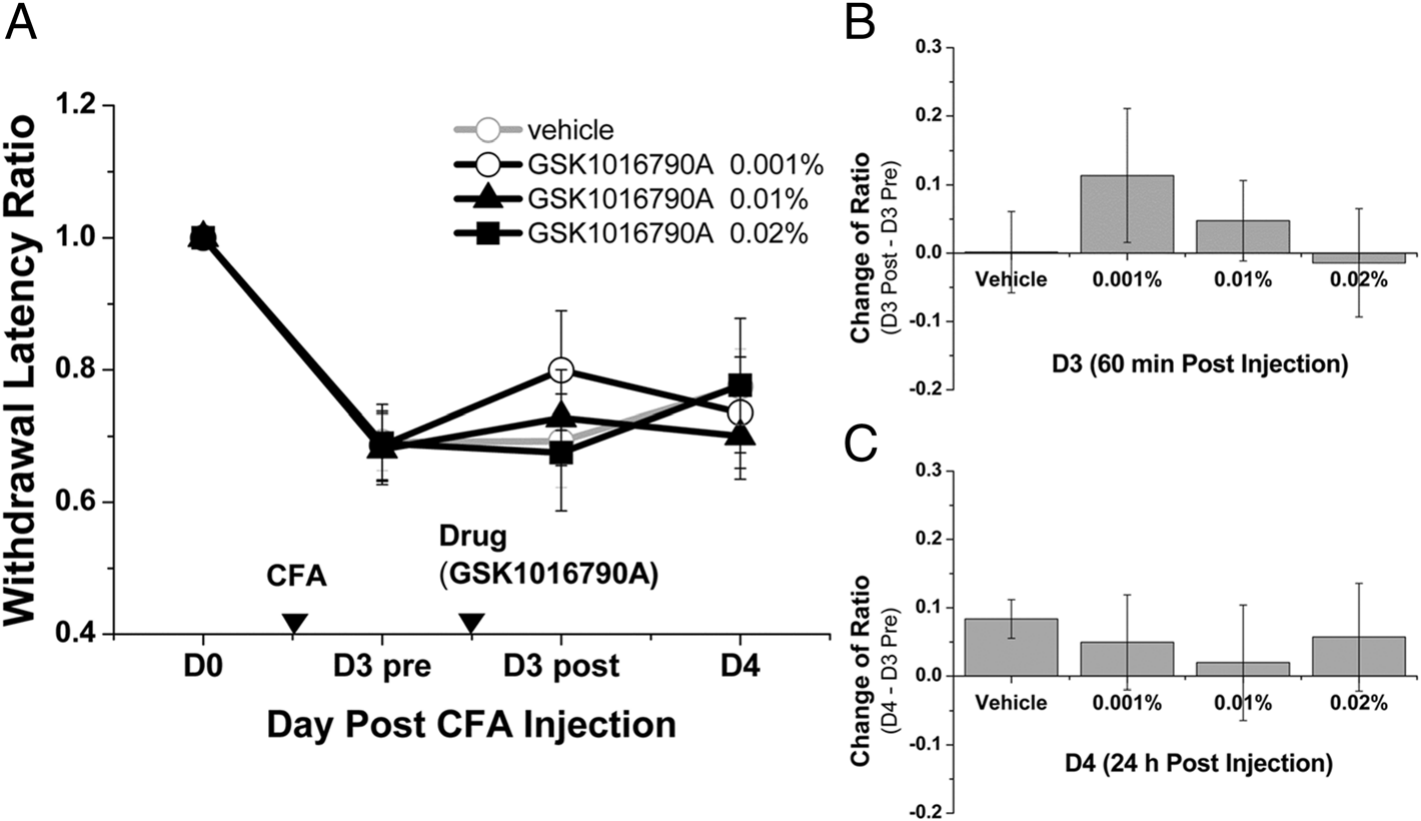
**TRPV4 agonist (GSK1016790A) injection at ST36 yielded no analgesic effect.** The behavioral test design was similar to that of MA; one difference was that GSK1016790A was injected at a different concentration and a volume of 10 μL into ST36. **(A)** Injections of different concentrations of GSK1016790A did not yield significant differences in within-group tests on D3 post and on D4. **(B and C)** No significant differences were found in between-group tests on D3 post and on D4. Within-group and between-group tests were designed similar to the MA behavioral test (Mann–Whitney rank sum test; *n* = 8–9). Data are means ± S.E.M.

For TRPV4, injection of the agonist, GSK1016790A, did not induce an analgesic effect (Figure 6A–C). Mice were grouped into the vehicle group and groups that received 0.001%, 0.01%, and 0.02% GSK1016790A. There were no significant differences in the withdrawal latency ratio among groups on D3 and D4 (Figure 6A). The change of ratio in between-group tests were not significant on D3 (Figure 6B) and D4 (Figure 6C).

**Figure 7:**
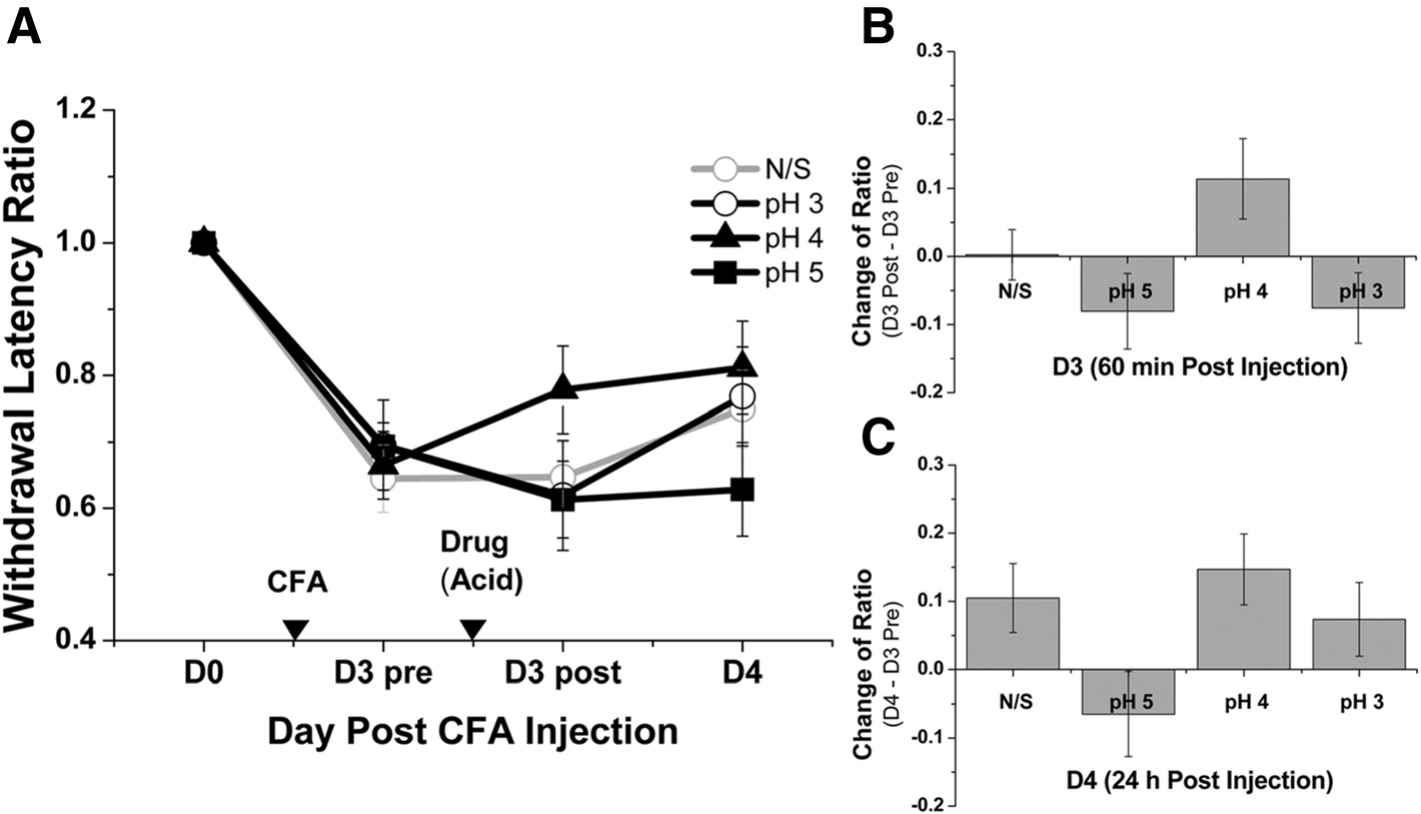
**ASIC3 agonist (acidified saline) injection at ST36 yielded no analgesic effect.** The behavioral test design was similar to that of MA; one difference was that acidified saline was injected at different pH values in a volume of 10 μL into ST36 only. **(A)** In within-group tests, injections of acidified saline at different pH values did not yield significant differences on D3 post and on D4. **(B and C)** No significant difference was observed between pH 7.4 normal saline (N/S) injection and acidified saline injection on D3 post and on D4. Within-group tests and between-group tests were designed similar to the MA behavioral test (Mann–Whitney rank sum test, *n* = 9–15). Data are means ± S.E.M.

Regarding ASIC3, acidified saline was used as a nonselective agonist and was injected into ST36 at pH 5, 4, and 3. No obvious analgesic effect was observed (Figure 7A–C). On D3 and D4, no significant differences were found in within-group comparisons (Figure 7A). Moreover, on D3 post (Figure 7B) and D4 (Figure 7C), the change of ratio in between-group tests were not significantly different between pH 7.4 normal saline injection and acidified saline injections.

As only capsaicin produced an analgesic effect similar to that of MA, the change of ratio between MA and 0.5% capsaicin injection were then compared. No significant difference was observed between the two treatments on D3 and D4, indicating that 0.5% capsaicin injection at ST36 was as effective as MA. These results suggest that the mechanosensitive TRPV1 is functional at ST36.

### Components of CWP were abundantly expressed at ST36

The questions remain as to why TRPV1 is abundantly expressed in non-neural cells and whether its expression in non-neural cells is related to acupuncture signaling to neurons. Furuya *et al.* stated that after mechanical stimulation of subepithelial fibroblasts of villi by food and water, these cells release ATP to the surrounding medium [32]. This elicits a CWP that activates neuronal terminals. Because CWP was reported during acupuncture in non-neural cells, [34] it is reasonable to speculate that non-neural cells at acupoints receive this mechanical signal via TRPV1 and subsequently release ATP after stimulation by acupuncture, as reported in other tissues [20,21]. ATP released by non-neural cells then stimulates nearby neurons via CWP. Although we cannot prove this via calcium imaging, we demonstrated this possibility by showing that the CWP components (pannexin 1, connexin 43, P2Y1, and P2Y2) were expressed in the anatomical layers with positive expression of TRPV1 in western blotting. Moreover, these components were abundantly expressed in ST36 compared to the sham point.

**Figure 8:**
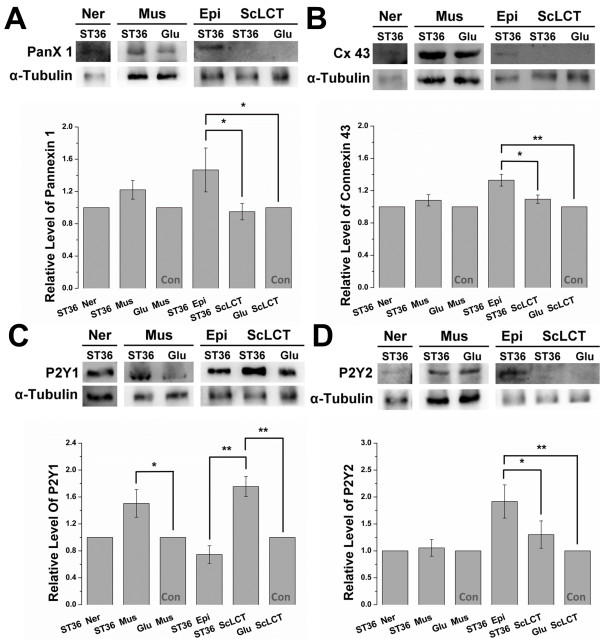
**Western blotting showed components for calcium wave propagation (CWP) were expressed abundantly at ST36.** Components tested were the ATP-releasing hemichannels pannexin 1 (PanX 1) and connexin 43 (Cx 43) and the purinergic receptors P2Y1 and P2Y2. Tissues from nerve (Ner), muscle (Mus), epimysium (Epi), and subcutaneous loose connective tissue (ScLCT) of ST36 or sham were collected in a manner similar to that described for mechanosensitive channel western blotting. **(A, B, C, and D)** Pannexin 1, connexin 43, P2Y1, and P2Y2 were expressed in nerve and muscle. **(A)** Pannexin 1 was more abundantly expressed at ST36 epimysium compared with ST36 ScLCT (*P* < 0.05) and sham ScLCT (*P* < 0.05). **(B)** Connexin 43 was more abundantly expressed at ST36 epimysium compared with ST36 ScLCT (*P* < 0.05) and sham ScLCT (*P* < 0.01). **(C)** P2Y1 was more abundantly expressed in ST36 muscle compared with the sham (*P* < 0.05) and in ST36 ScLCT compared with ST36 epimysium (*P* < 0.01) and sham ScLCT (*P* < 0.01.) **(D)** P2Y2 was more abundantly expressed at ST36 epimysium compared with ST36 ScLCT (*P* < 0.05) and sham ScLCT (*P* < 0.01). All four components were expressed in muscle and the epimysium of ST36. These two layers also abundantly expressed TRPV1. The relative levels of target proteins were calculated in a manner similar to that used for western blotting of mechanosensitive channels (*P < 0.05, **P < 0.01; Mann–Whitney rank sum test; pannexin 1, *n* = 8–12; connexin 43, *n* = 6; P2Y1, *n* = 5–9; P2Y2, *n* = 7). Data are means ± S.E.M.

The results showed that pannexin 1, connexin 43, P2Y1, and P2Y2 were expressed in nerve, muscle, and connective tissue. Furthermore, pannexin 1 was abundantly expressed in ST36 epimysium compared with sham ScLCT (1.47 ± 0.27-fold, *P* < 0.05) and ST36 ScLCT (1.47 ± 0.27 vs. 0.95 ± 0.10, *P* < 0.05, Mann–Whitney rank sum test) (Figure 8A). Similarly, connexin 43 was abundantly expressed at ST36 epimysium compared with sham ScLCT (1.33 ± 0.07-fold, *P* < 0.01) and ST36 ScLCT (1.33 ± 0.07 vs. 1.09 ± 0.05, *P* < 0.05) (Figure 8B). ST36 muscle exhibited abundant P2Y1 expression compared with the sham (1.50 ± 0.20-fold, *P* < 0.05). Moreover, abundant P2Y1 expression was found at ST36 ScLCT compared with ST36 epimysium (1.76 ± 0.15 vs. 0.74 ± 0.13, *P* < 0.01) and sham ScLCT (1.76 ± 0.15-fold, *P* < 0.01) (Figure 8C). Abundant P2Y2 expression was observed at ST36 epimysium compared with ST36 ScLCT (1.92 ± 0.31 vs. 1.30 ± 0.25, *P* < 0.05) and sham ScLCT (1.92 ± 0.31-fold, *P* < 0.01) (Figure 8D).

The occurrence of CWP requires the expression of either the pannexin 1 or connexin 43 hemichannels in an anatomical layer for ATP release, and expression of the P2Y1 or P2Y2 purinergic receptors for ATP signaling. After cross-matching the expression patterns of the components, only muscle and epimysium other from neuron expressed both hemichannels and purinergic receptors. These two layers were also the site at which TRPV1 was abundantly expressed. This expression pattern made it spatially possible for TRPV1 to pass on the ATP signal and trigger CWP to neurons.

## Discussion

After localizing functional ST36, this study showed that TRPV1, TRPV4, and ASIC3 were abundantly expressed in different anatomical layers of this acupoint. Furthermore, histological results revealed that, not only in nerve fibers, the channels were expressed in skeletal muscle cells and possibly expressed in fibroblasts. The injection of agonists of channels into ST36 showed that only capsaicin replicated acupuncture analgesia. TRPV1 expression in nerves is understandable. Conversely, the abundant expression of TRPV1 in non-neural tissues at ST36 was tried to be explained. After TRPV1 activation, ATP may be released by non-neural cells and trigger CWP, which indirectly conveys signals to nearby neurons. This possibility was confirmed by showing abundant expression of the components of CWP in the anatomical layers of ST36 that also abundantly expressed TRPV1.

The results of immunofluorescence showed that all three channels were expressed in muscle fibers. This was in accordance with previous findings that TRPV1, [49,50] TRPV4, [45,51] and ASIC3 [52] are expressed in skeletal muscle. The microscopic slides of immunofluorescence accidentally discovered higher expression of TRPV1 at the muscle margin. This is rather interesting because the acupuncture sensation (de-qi) is stronger just after needle-tip insertion into perimuscular fascia (epimysium) [42]. However, this warrants confirmation. The slides also showed that these channels were expressed in cells of the epimysium and subcutis. TRPV1, [44] TRPV4, [45,46] and ASIC3 [47,48] are reported to express in fibroblast, which is the primary cell in connective tissue; thus, it is suggestive that the positive cells in the epimysium and subcutis are fibroblasts. If so, mechanosensitive channels may participate in the connective tissue theory proposed by Langevin *et al*. [53-55].

This study also demonstrated an acupuncture-like analgesic effect of capsaicin injection at ST36. This is also true in clinical settings, as others reported the acupuncture-like effect of the topical application of capsaicin on acupoints [56,57]. Capsaicin can cause conduction analgesia (or desensitization) [58,59]. However, this happens only when capsaicin is delivered to the innervating nerve and functions as lidocaine as conduction blocker. Nonetheless, the deep peroneal nerve innervates ST36 [60,61]. In contrast, the tibia nerve (partiallly the sural and saphenous nerves) innervate the inflamed and tested paws. Anatomically, conduction analgesia on the deep peroneal nerve would impair function of ST36 but have little effect on nerves that innervate the hind paw. Moreover, the deep peroneal nerve is separated from the tibia nerve by an interosseous membrane. Leakage was unlikely because the injection depth was insufficient to penetrate the membrane and the injected volume was minimized to 10 μL. Also, it has been proven that stimulation but not inhibition of peroneal nerve would result in analgesic effect to nociception [60,61]. It is more logical to assume that analgesia was caused by a mechanism other than conduction analgesia.

Capsaicin has been used in other experiments to study noxious stimulus-induced analgesia (NSIA) [62,63]. Capsaicin stimulation releases glutamate and substance P at the spinal dorsal horn, activating NMDA, AMPA/kainate, mGluR5, and NK1 receptors on inhibitory interneurons and releases GABA or glycine to provide an analgesic effect. In addition to the spinal cord, supraspinal activation of the nucleus accumbens was reported, which turns on the opioid-related descending inhibitory pathway and induces heterosegmental antinociception [40,64,65]. The NSIA mechanism greatly overlaps the proposed acupuncture analgesia mechanism [14-17] and both involve similar spinal and supraspinal regulation. This may explain why capsaicin injection at ST36 replicates MA analgesia. However, capsaicin was also injected into the sham point, but with no analgesic effect. There are several logical reasons for this: a nerve trunk (deep peroneal nerve) is closer to ST36 and may result in greater stimulation; nerves supplying acupoints are enriched for TRPV1; [66] the non-neural layers in our experiment expressed more TRPV1. Higher TRPV1 expression may lead to more ATP release in ST36, [19] suggesting greater purinergic P2X receptor (P2X3 or P2X2) activation that also contributes to NSIA [67,68]. Therefore, we suggest that capsaicin injection at ST36 induces greater TRPV1 and P2X receptor activation and results in better NSIA than that at the sham point.

TRPV1 is a polymodal receptor because it can be activated by various types of stimulation including capsaicin, heat (>43°C), low pH, voltage, and endogenous lipids [69]. As acids can also activate TRPV1, it is worth asking why the acidified saline injection did not activate TRPV1 and provide analgesic effects, similar to capsaicin. We reviewed literature regarding EC50 of capsaicin and acid for TRPV1. Under in vitro conditions at 37°C, EC50 for capsaicin at pH 7.4 is 640 nM and EC50 for acid stimulation is pH 5.35 [70]. The ratio for capsaicin used over capsaicin’s EC50 is approximately 2.6 × 10^4^, whereas the ratio for acid saline used over its EC50 is much smaller (2.2–2.2 × 10^2^). This explains why even saline at pH 3 was not sufficient to cause NSIA by TRPV1.

One may ask why TRPV4 and ASIC3 injections did not yield analgesic effects similar to TRPV1. This could be because of the differences in permeability to calcium that acts as modulating messenger. ASIC3 is permeable to sodium, but not calcium, in physiological conditions. TRPV4, although permeable to both cation, is less permeable to calcium than to TRPV1 [71]. This may also be because of differential expression of channels on nerve fibers. TRPV1 is mostly expressed in C-fibers and Aδ-fibers, whereas TRPV4 and ASIC3 are not restricted to them [5,72,73]. As different fiber types terminate in various spinal lamina, it is reasonable to think that a different effect was introduced.

Our group previously reported that TRPV1, TRPV4, and ASIC3 are upregulated in DRGs after inflammatory pain but attenuated after electroacupuncture [4,5]. Balance recovery is emphasized in traditional Chinese medicine, having hyper-activated to down-regulate and hypo-activated to up-regulate. TRPV1 upregulation observed during pain may serve to enhance NSIA and restore balance.

**Figure 9:**
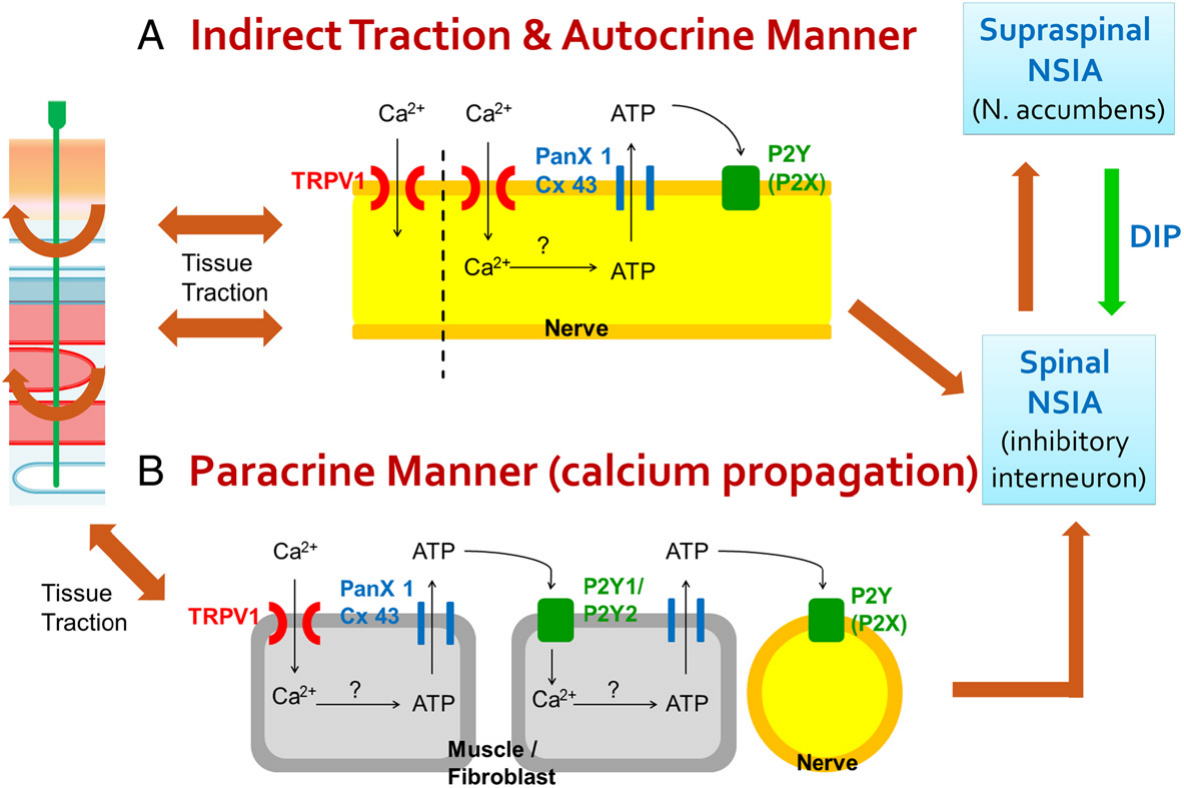
**Schematic diagram of the proposed interface between mechanostimulation and biological signaling at acupoint.** Manual acupuncture at acupoints causes tissue traction during manipulation and results in the activation of mechanosensitive TRPV1 on the cell membrane. This leads to two parallel sensing pathways: the neural and the non-neural cell initiated sensing pathways. **(A)** In the former, TRPV1 of nerves is stimulated after traction, which generates an action potential after channel opening. It is also possible that increased intracellular calcium leads to ATP release by hemichannels to the extracellular matrix (ECM) after TRPV1 stimulation. The released ATP acts in an autocrine manner and results in self-stimulation (neuron) by purinergic receptors (P2Y or P2X). **(B)** In the latter, TRPV1 on muscle fibers or fibroblasts is activated and increases calcium influx leading to ATP release to the ECM. The released ATP then activates purinergic receptors on nearby cells (another muscle fiber or fibroblast). This increases intracellular calcium again and another ATP is released. The chain-like paracrine process of ATP release and calcium signaling is named calcium wave propagation (CWP). As in other circumstances, non-neural cells can pass on message to neurons via CWP after traveling for a certain distance. The occurrence of antinociceptive regulation requires that these pathways activate noxious stimulus-induced analgesia (NSIA), either spinally (by inhibitory interneurons) or supraspinally [by the nucleus accumbens and descending inhibitory pathway (DIP)]. This hypothesis is supported by previous reports: ATP release during acupuncture (Goldman et al. [19] and Takano et al. [18]); CWP during acupuncture in non-neural cells (Li et al. [34]); signaling from non-neural cells to neurons via CWP (Furuya et al. [32] and Koizumi et al. [33]); and numerous reports on CWP and NSIA. Abbreviations: pannexin 1 (PanX 1); connexin 43 (Cx 43); noxious stimulus-induced analgesia (NSIA); descending inhibitory pathway (DIP).

The question remains as to how TRPV1 activation is connected to nerve stimulation. This seems obvious if TRPV1 activation occurs after direct puncturing of the membrane of nerve branches, which generates an action potential. However, in clinical practice, direct puncturing of nerve branches is avoided to prevent potential nerve injury. Therefore, it is more likely that nerve stimulation occurs indirectly. According to Langevin *et al.*, acupuncture causes local tissue displacement [53-55]. It is conceivable that local traction by displacement transfers physical forces to nerves and activates TRPV1 to generate an action potential. Alternatively, TRPV1 expression in nerves results in ATP release, [20,21] which stimulates self-purinergic receptors in an autocrine manner (Figure 9A). ATP may be released by muscle fibers or fibroblasts. Released ATP then conveys the signal to nerves by CWP, as demonstrated by Furuya *et al.* in villi mechanotransduction and by Koizumi *et al.* in keratinocyte mechanotransduction [32] (Figure 9B). The involvement of CWP is highly likely because the occurrence of CWP in non-neural cells during acupuncture has been reported [34]. Furthermore, similar to TRPV1, pannexin1, [74,75] conexxin43, [75-79] P2Y1, [32,80-82] and P2Y2 [74,82-84] are been reported to express in muscle and fibroblast. Also, from the western blotting of this study, TRPV1 and the CWP components were all expressed in muscle and epimysium layers. These evidences increase the likelihood that CWP carried on the signaling after TRPV1-related ATP release. CWP participation during acupuncture may explain why acupuncture meridians (or channels) did not fully match the anatomy of nerve innervation. CWP may bridge the gap between the two. Moreover, ATP responding P2X receptors also participate in NSIA; [67,68] thus, CWP may result in increased P2X receptor recruitment and NSIA amplification.

There were several limitations in this study. A nonselective acidified saline injection was used to activate ASIC3 because of limitations on the acquisition of commercial ASIC3-selective agonists. The pH value was selected considering that ASIC3 is majorly involved in pH 4 saline-induced chronic muscle pain [41]. The role of ASIC3 during acupuncture would have been better explained if an ASIC3 selective agonist was available. Moreover, directly block of the MA analgesic effect with antagonists of mechanosensitive channels was not performed. Most antagonists are already pain relieving when systemically administered, [41,85-88] and local injections of antagonists prior to acupuncture may be too damaging for the acupoint. Nonetheless, replicating the MA analgesic effect by injecting capsaicin into acupoint ST36 may provide knowledge on the functional role of TRPV1 at acupoints.

## Conclusions

As a conclusion, TRPV1 is expressed in neural and non-neural cells at acupoints, and its activation may replicate the effect of acupuncture. Also, both the neural cell initiated sensing pathways and the non-neural cell initiated sensing pathways, which involves calcium wave propagation, may participate in conveying signal from mechanostimulation to nervous system. These results may lead to clinical studies of capsaicin application to acupoints. Perhaps the application of capsaicin or other TRPV1 agonists will represent an additional treatment option and enhance the effect of acupuncture. Given the capabilities of TRPV1 for receiving mechanical and thermal stimulations, as in acupuncture and moxibustion (thermal stimulation in traditional Chinese medicine), more extensive studies on the role of TRPV1 during acupuncture should be performed to determine whether it is an “acupuncture-responding channel”.

## Abbreviations

TRPV1: Transient receptor potential vanilloid receptors 1; TRPV4: Transient receptor potential vanilloid receptors 4; ASIC3: Acid-sensing ion channel 3; CWP: Calcium wave propagation; MA: Manual acupuncture; EA: Electroacupuncture; CFA: Complete Freund’s adjuvant; Glu: Gluteus maximus sham point; D0: Day 0; D1: Day 1; D2: Day 2; D3: Day 3; D3 pre: Day 3 before intervention; D3 post: Day 3 after intervention; EC50: Half-maximal effective concentration; Ner: Deep peroneal nerve; ScLCT: Subcutaneous loose connective tissue; Epi: Epimysium; Mus: Muscle; PanX 1: Pannexin 1; Cx 43: Connexin 43; NSIA: Noxious stimulus-induced analgesia; DIP: Descending inhibitory pathway.

## Competing interests

The authors declare that they have no competing interests.

## Authors’ contribution

SYW conceived the study, carried out the experiments, analyzed the data, and wrote the manuscript. SYW and WHC participated in designing the experiments. YWL involved in drafting the manuscript and gave final approval of the submitted version. CLH involved in critical revision of important intellectual contents. All authors read and approved the final manuscript.

## Pre-publication history

The pre-publication history for this paper can be accessed here:

http://www.biomedcentral.com/1472-6882/14/96/prepub
